# Multiple Mineralocorticoid Response Elements Localized in Different Introns Regulate Intermediate Conductance K^+^ (Kcnn4) Channel Expression in the Rat Distal Colon

**DOI:** 10.1371/journal.pone.0098695

**Published:** 2014-06-05

**Authors:** Bryan O’Hara, Diego Alvarez de la Rosa, Vazhaikkurichi M. Rajendran

**Affiliations:** 1 Department of Biochemistry, West Virginia University School of Medicine, Morgantown, West Virginia, United States of America; 2 Department of Physiology, University of La Laguna, Tenerife, Spain; University of Florida, United States of America

## Abstract

An elevated plasma aldosterone and an increased expression of the intermediate conductance K^+^ (IK/Kcnn4) channels are linked in colon. This observation suggests that the expression of Kcnn4 gene is controlled through the action of aldosterone on its cognate receptor (i.e., mineralocorticoid receptor; MR). In order to establish this, we performed chromatin immunoprecipitation (ChIP) assay to identify the MR response elements (MREs) in a region that spanned 20 kb upstream and 10 kb downstream of the presumed transcription start site (TSS) using chromatin from the colonic epithelial cells of normal and aldosterone-treated rats. MREs were immunoprecipitated in an approximately 5 kb region that spanned the first and second introns in the aldosterone rats. These regions were individually cloned in luciferase-expression vector lacking enhancer activity. These clones were tested for enhancer activity in vitro by transfecting in HEK293T and CaCo2 cells with MR and aldosterone treatment. At least four regions were found to be responsive to the MR and aldosterone. Two regions were identified to contain MREs using bioinformatics tools. These clones lost their enhancer activity after mutation of the presumptive MREs, and thus, established the functionality of the MREs. The third and fourth clones did not contain any bioinformatically obvious MREs. Further, they lost their activity upon additional sub-cloning, which suggest cooperativity between the regions that were separated upon sub-cloning. These results demonstrate the presence of intronic MREs in Kcnn4 and suggest a highly cooperative interaction between multiple intronic response elements.

## Introduction

The mammalian colon plays a critical role in regulating K^+^ homeostasis, as it displays both active K^+^ absorption and active K^+^ secretion [1]. An ATP-dependent electroneutral H-K exchange (i.e., H, K-ATPase) localized on the apical membranes regulates active K^+^ absorption, while K^+^ channels also situated in these membranes mediate active K^+^ secretion in the colon [2], [3]. The Ca^2+^-activated large conductance K^+^ (Kcnma1) and intermediate conductance K^+^ (Kcnn4) channels have been identified as the major K^+^ channels present in the colonic epithelial cells [4]. The apical Kcnma1 K^+^ channels [characterized as iberiotoxin (IbTX)-sensitive] have been shown to be responsible for active K^+^ secretion, while the basolateral Kcnn4 K^+^ channels [characterized as clotrimazole (CLT)-sensitive] have been hypothesized to maintain the cell hyper-polarization that provides the driving force for the agonist (cAMP/Ca^2+^)-induced Cl^−^ secretion [4], [5]. Although Kcnma1 channels have been localized to only apical membranes, Kcnn4 channels are localized on both the apical and basolateral membranes of the colon [6]. In recent studies, Kcnn4 splice variants, Kcnn4c and Kcnn4b, are shown to encode the apical and basolateral IK channels, respectively [7].

Aldosterone, a mineralocorticoid hormone plays a critical role in regulating colonic K^+^ movement [8]. Aldosterone stimulates both active K^+^ absorption and active K^+^ secretion in the rat distal colon [4]. In recent studies, we have found that aldosterone induces active K^+^ secretion by enhancing the mucosal expression of Kcnma1 and Kcnn4c specific proteins and that this occurs at least in part at the transcriptional level [9]. Aldosterone is responsible for maintaining Na^+^ homeostasis, water retention and blood pressure, and regulates its physiological function through the mineralocorticoid receptor (MR) [10]. MR can be grouped with the androgen, glucocorticoid and progesterone receptors, in that each recognizes largely the same hormone response element (HRE). The HRE is composed of an inverted repeat separated by three nucleotides, which generally appears as the sequence AGAACAnnnTGTTCT [11], [12], [13], [14]. The hormone-bound nuclear receptor (NR) interacts at the HRE through a coregulator with the basal transcription initiation complex, transcription factors, chromatin remodeling proteins, and histone acetylases and methylases to activate transcription [15], [16]. The HREs can be localized within the proximal promoter and can also be many kilobases (kb) upstream or downstream of the transcription start site (TSS). The aldosterone responsiveness of Kcnn4 could be the result of the direct action of MR on the gene or indirectly by activation of a function which itself activates Kcnn4 expression. Thus, by determining that Kcnn4 is aldosterone-responsive, we sought to demonstrate that this occurs as a direct result of the MR action by scanning the environs of the Kcnn4 TSS for the MR response elements. Our results revealed that the MR exerts long-range regulation of Kcnn4 through distal sites that are located within the body of the gene.

## Materials and Methods

### Animals

The rats demonstrating high levels of aldosterone were produced by feeding Na-free rat chow (MP Biochemicals, Solon, OH) to normal male Sprague-Dawley rats (201 – 225 g) for 6 - 7 days. The Na-free diet enhanced the serum aldosterone levels by 15-fold [9], [17]. The animals fed with the Na-free diet are referred to as aldosterone animals. The rats that were fed a regular rat chow are referred to as normal animals. All of the animals were given food and water ad libitum. The experimental protocols used in this study were approved by the West Virginia University Institutional Animal Care and Use Committee. Animals were anesthetized with 40 mg/kg pentobarbital given intraperitoneally (*i.p*). Following the removal of the colon, the animals were euthanized using a high dose of pentobarbital (100 mg/kg).

### Chromatin Immunoprecipitation (ChIP) Assay

ChIP assay was performed essentially as described by Peng et al [18]. In brief, the distal colons excised from the anesthetized rats were flushed with ice cold saline. The colons were then incubated at 4°C in 5 mM Hepes-Tris buffer (pH 7.5) containing 30 mM NaCl, 5 mM EDTA and 0.5 mM DTT for 30 min to loosen the epithelial cells from the muscular layers. The loosened epithelial cells were collected by palpitation and centrifugation and then washed once in a cold PBS prior to being resuspended into a 20 ml ice cold PBS. The cell pellets divided into 0.5 ml aliquot was cross-linked with 1% formaldehyde for 10 min at 37°C with shaking. The cross-linking stopped with the addition of glycine at 0.125 M. The cells were resuspended in a 5 ml lysis buffer and then sonicated to disrupt chromatin, yielding a DNA product of approximately 3 kb (not shown). The protein concentration of clarified chromatin was determined using the Pierce BCA protein assay kit. The input DNA was purified from 100 µg of lysate. For immunoprecipitation, 1 mg of lysate was precleared by incubation with Protein A/G agarose beads pre-blocked with BSA and salmon sperm DNA. One mg of lysate was incubated overnight with 5 µg of either the MR-specific antibody SC-11412 or a normal rabbit IgG SC-2027 (Santa Cruz Biotechnology). The immunoprecipitates were gathered on pre-blocked Protein A/G agarose beads, and then washed and eluted. The cross-links were reversed for 18 hrs at 65°C and the DNA was purified using the Qiaquick PCR Purification Kit (Qiagen).

### Real Time Polymerase Chain Reaction (RT-PCR)

The primer pairs used for the amplification of the approximate 100 bp products were designed using the REALTIME PCR program of Integrated DNA Technologies. The PCR products were derived from approximately 1 kb blocks and ranged from 20 kb upstream (pair number 1) to 10 kb downstream (pair number 29) of the presumed TSS as determined from the longest extant rat mRNA (NCBI reference sequence AF156554) [6]. The immunoprecipitated DNA was amplified on the Bio-Rad iCycler, and the amplification was monitored using a RT^2^ Real-Time SYBR Green/Fluorescein PCR kit (SA Biosciences). The threshold cycle (Ct) values were calculated in the PCR Baseline Subtracted Mode according to the Bio-Rad instructions. The differential recovery was calculated by comparing the Ct values for the specific antibody to the control antibody and was presented as a fold-enrichment over the control antibody. The input DNAs were included to verify accurate PCR product sizes, and to ensure the primers worked efficiently and would not be directed towards the repetitive DNA. All of the products were analyzed through melt curves and by gel electrophoresis to confirm the validity of the Ct values. The primer pairs used in ChIP assays are listed in Table S1.

### Plasmid Construct

The candidate enhancer regions were generated by PCR with primers containing HindIII linkers and then cloned in the HindIII site of the pGL4.23 [*lic2/minP*] vector (Promega). The primer sequences are provided in the Table S2. The pGL4.23 has a minimal promoter, which direct transcription of the firefly luciferase gene and is suitable for detecting activity of the inserts within the enhancer activity. Both the loop-outs and mutations were made using the QuikChange Mutagenesis Kit and processes according to the manufacturer’s instructions (Stratagene Cloning Systems). All of the plasmids were verified by sequencing. The sequences of the inserts have been provided in the Figure S1. In addition, the sub-clones were made with overlaps (up to 50 bp) to ensure there was not any simple destruction of the regulatory site. The positions of the inserts were determined from the Rat Nov. 2004, Baylor 3.4/rn4 assembly, University of California Santa Cruz Genome Browser [19], [20]. Clone-1∶79614936–79616822, clone-1C: 79616163–79616822, clone-3A: 79614936–79615624, clone-3B: 79615577–79616212, clone-5∶79618558–79619893, clone-5A: 79618558–79619033, clone-5B: 79618981–79619526, clone-5Ba: 79618981–79619296, clone-5Bb: 79619276–79619526, clone-5C: 79619456–79619893, clone-6∶79619756–79621314, clone 6A 79620797–79621315, clone 6B 79620252–79620854, clone-6C: 79619756–79620271 (all the positions numbered refer to rat chromosome 1).

### Cell Culture, Transfection and Luciferase Activity

The HEK293T (Human Embryonic Kidney cell line; Thermo Scientific HCL4517), CHOK1 (Chinese Hamster Ovary cell line; Sigma-Aldrich 85051005), and CaCo2 (Colorectal adenocarcinoma cell line; ATCC HTB-37) cells were grown in DMEM (17–205-CV, Mediatech) with 100 IU/ml penicillin, 100 µg/ml streptomycin (MT-30-002-CI, Mediatech), glutamine (SG-200, HyClone) and 10% FBS (SH 309 1003, Thermo Scientific). The cells were plated at a 2×10^4^ cells/well in 96-well plates. One day after plating, the cells were washed once with PBS and transfected with either a mixture of pGL4.23 vector with (clones) or without (pGL4.23) the candidate enhancer inserts. The HEK293T cells were transfected with the pGL4.73 (renilla luciferase was under the control of a strong SV40 promoter) and either the pCDNA3.1 (control) or the pCDNA3.1-hMR (generous gift of Drs. Ronald M. Evans, The Salk Institute for Biological Studies, La Jolla, California, USA; and Peter Fuller, Prince Henry’s Institute, Clayton, Victoria, Australia) were grown in a growth medium with 5% charcoal-dextran-stripped FBS (100–119, Gemini Bio-Products). Next, the cells were fed with the same medium with or without aldosterone (A9477, Sigma) or cortisol (S1696, Selleckchem) on day 2. The luciferase activity was measured on day 3 using the Dual-Luciferase Reporter Assay System (Promega, Madison, WI, USA) and according to the manufacturer’s instructions. The positive control used was the MMTV-luc, which expresses the firefly luciferase under the control of the steroid-responsive mouse mammary tumor virus promoter. The control routinely presented as greater than a 30-fold enhancement with MR transfection and aldosterone or cortisol (data not shown).

### ^35^S-methionine Labeling/Western Blotting

The ^35^S-methionine labeling was performed as described by Arteaga et al [21]. In brief, the CHOK1 cells transfected with either the control or the MR-containing plasmid, then they were incubated in the growth medium that was free of the methionine containing ^35^S-methionine (100 µCi/ml). Following a 24 h incubation period the cells were washed and homogenized in a PBS. The homogenate was used for immunoprecipitation with the SC-11412 antibody (MR antibody). The precipitate was processed by SDS-PAGE and the dried gel that was exposed to the X-ray film was developed. Western blotting was performed as described earlier [9]. Furthermore, the rat colonic epithelial cell protein that was extracted from the aldosterone treated rat was immunoprecipitated with the control antibody or the SC-11412. The precipitated protein resolved on SDS-PAGE electrophoresis was transferred to the nitrocellulose membrane and was probed with the SC-11412 as primary antibody, and followed by goat anti-rabbit HRP (Santa Cruz Biotechnology SC-2004). The blot was developed using the ECL+Plus kit (GE Healthcare).

### Statistical Analyses

GraphPad Prism 4.0 computer software was used for the statistical analysis, utilizing the Student’s unpaired two-tailed *t* test, with *p*<0.05 being viewed as significant.

## Results

### Validation of the Anti-MR Antibody (SC-11412) and Identification of MR in the Rat Colonic Epithelial Cells

**Figure 1:**
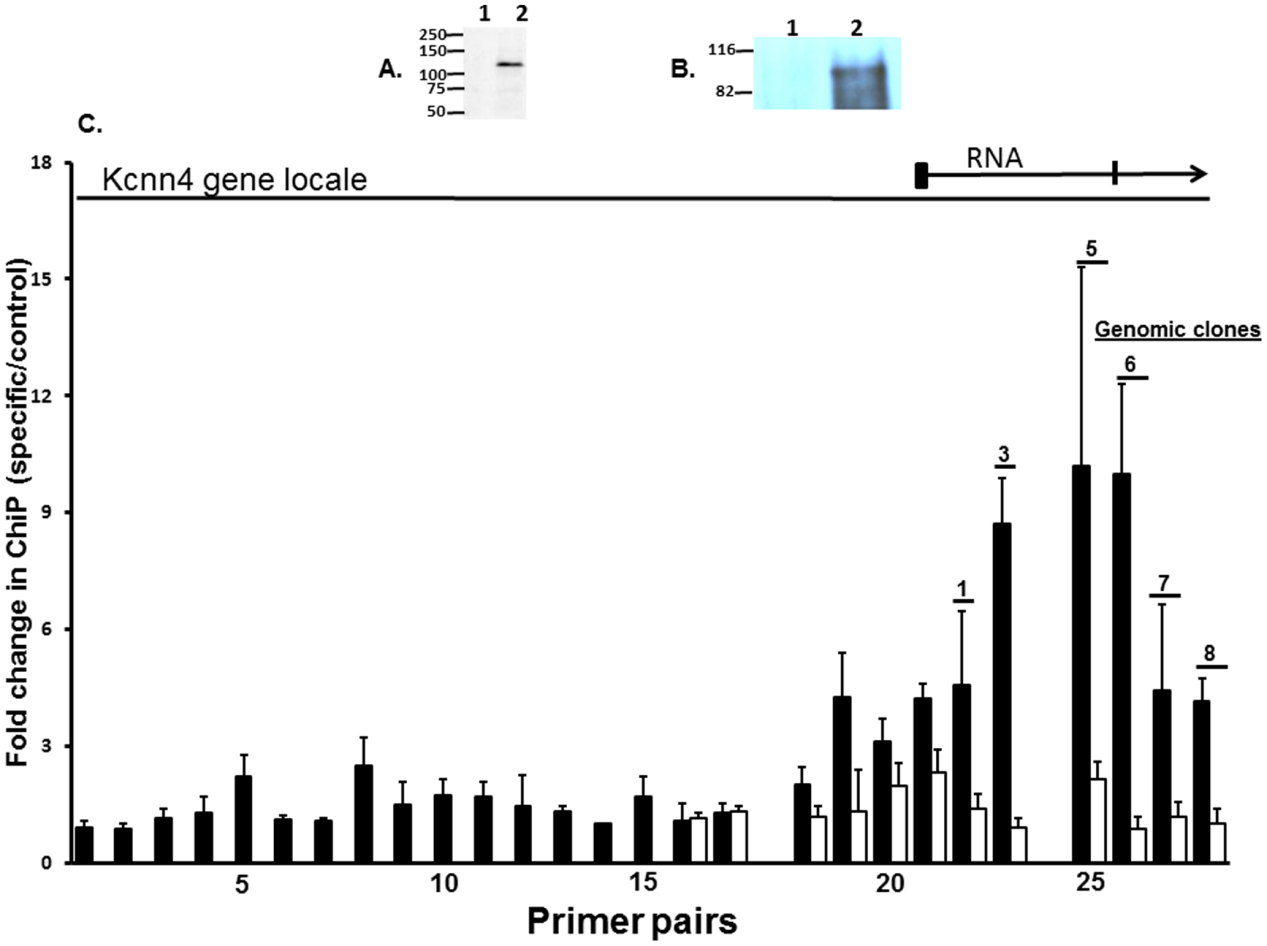
Characterization of anti-mineralocorticoid receptor antibody (SC-11412) and its use in chromatin immunoprecipitation (ChIP) assay. [**A**] *Characterization of SC-11412 antibody*: The CHOK1 cells were transfected with vector (**lane-1**) or the vector containing a mineralocorticoid receptor (MR) (**lane-2**), labeled with ^35^S-methionine, immunoprecipitated with SC-11412 antibody to MR, resolved on SDS-PAGE, and the gel used to expose X-ray film. [**B**] *Immunoprecipitation of MR-like proteins from rat colonic epithelial cells*: Protein extracts of the colonic epithelial cells from an aldosterone rat was immunoprecipitated with the control (**lane-1**) or the SC-11412 (**lane-2**) antibody. The immnoprecipitated proteins were resolved on SDS-PAGE and transferred to the nitrocellulose membrane and probed with the SC-11412 antibody. [**C**] *ChIP assay of MR*: Numbers on the X-axis represent primer pairs directed to 1 kb blocks starting at approximately 20 kb upstream (number-1) of the Kcnn4 presumed transcript start site (TSS) and extending to 10 kb downstream (number-29) of the presumed TSS (located in block 21). The Y-axis represents fold enrichment in the RT-PCR comparing the specific to the control antibody. Data presented represents mean ± SE of 4 independent experiments using distal colon epithelial cells from aldosterone (**closed bars**) and normal rats (**open bars**). The RNA transcript is indicated by an arrow. The presumed promoter region is immediately to the left of the arrow. Exons 1 and 2 are indicated by blocks. Genomic regions cloned in pGL4.23 are indicated by bars and are numbered.

In order to characterize the SC-11412 anti-MR antibody as being specific and potent, the CHOK1 cells expressing the hMR were labeled with ^35^S-methionine and the protein extracts were immunoprecipitated with either the control antibody or SC-11412. The immunoprecipitate was run on an SDS-PAGE gel and used to expose the X-ray film. A single band corresponding to the expected size of the full protein (107 kDa) [22] was detected (Figure 1A). To determine whether the MR was present in the aldosterone rat colonic epithelium, an extract of this was made, immunoprecipitated with control antibody or SC-11412, and then examined by a western blot using SC-11412 as the primary antibody. A primary protein band of approximately 107 kDa was detected only in the SC-11412 precipitate (Figure 1B). A smearing was also detected, which we presumed to be the result of the partial protein degradation. These results indicated that SC-11412 was suitable for use in chromatin immunoprecipitation to examine the interaction of the receptor and chromatin.

### Chromatin Immunoprecipitation with Antibody to MR Suggests the Presence of MREs within the Kcnn4 Gene

We took the position 79613736 on chromosome 1, the start of the longest extant cDNA clone, as the presumed TSS. Given that all eight of the other cDNAs with the entire coding region (UCSC Genome Browser) starts downstream of position 79613736, this position is very likely to be close to the TSS for Kcnn4. The primer pairs were designed to 1 kb blocks of DNA extending 20 kb upstream and 10 kb downstream of the presumed TSS. The ChIP assays were performed using the SC-11412 antibody and the control antibody against colonic epithelial chromatin from the aldosterone rats, in which the binding of MR to its MREs was expected to occur in the neighborhood of induced genes. The ChiP assays were also performed on the colonic epithelial cells of the normal rat colon. The primers were then used to assess the enrichment of specific areas in the RT-PCR. The immunoprecipitation of the SC-11412 antibody, comparison to the control antibody, suggests the presence of MREs in a broad area spanning introns 1 and 2 of Kcnn4 in the aldosterone rats (Figure 1C). In contrast, the normal rats did not show any evidence of enrichment of this region.

### Functional Assays of Clones with Candidate Enhancer

**Figure 2:**
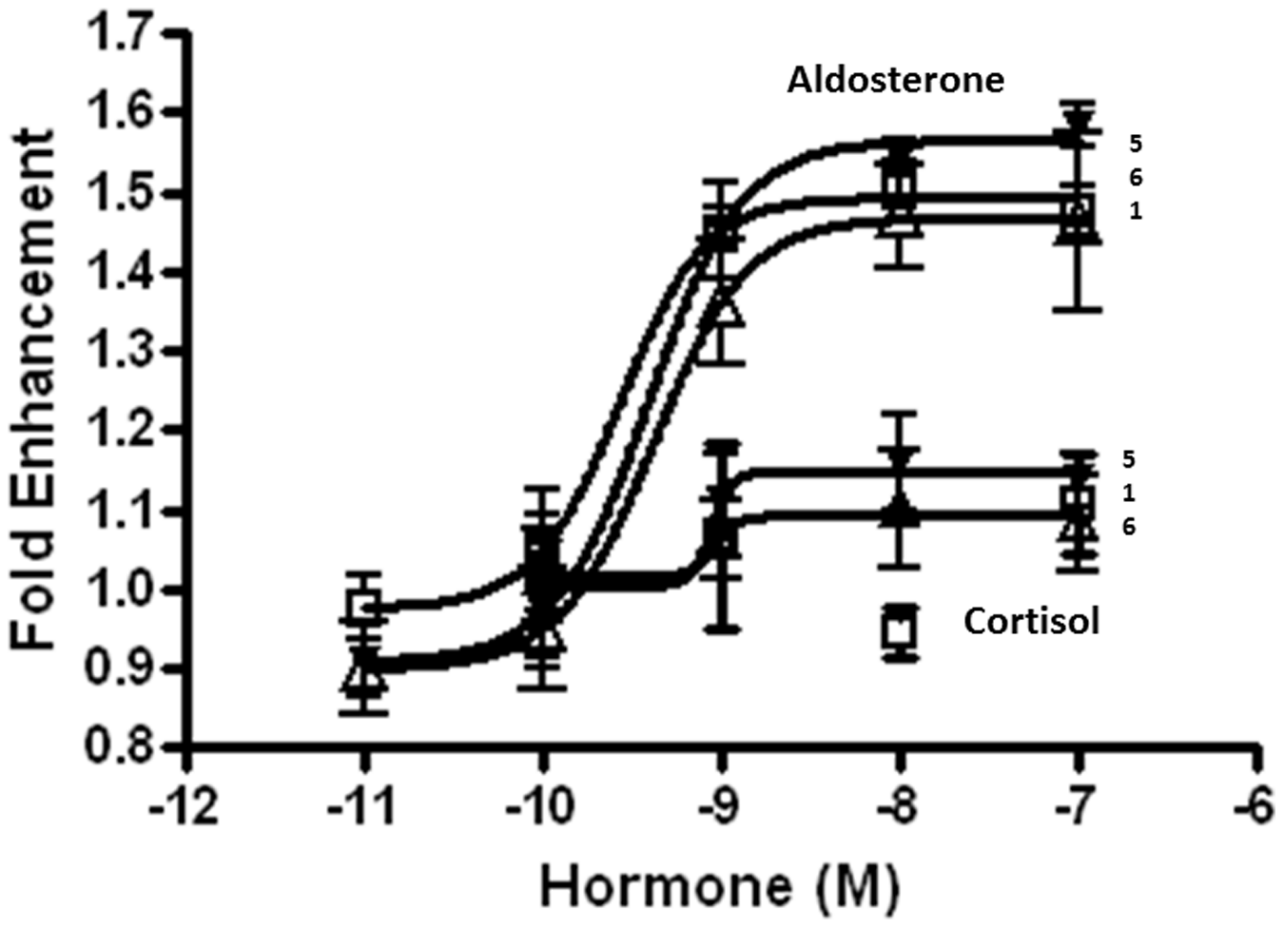
Effect of mineralocorticoid receptor (MR) and aldosterone in HEK293T cells expressing Kcnn4 genomic clone. Regions of the Kcnn4 genes showing positive response in a ChIP assay and indicated by bars in Figure-1C (clone-1, clone-3, clone-5, clone-6, clone-7 and clone-8) were sub-cloned in pGL4.23 and tested for the presence of MR/aldosterone (mineralocorticoid)-responsive enhancers (MRE). The HEK293T cells were transfected with pGL4.23 or pGL4.23 containing Kcnn4 genomic clones. The cells transfected with Kcnn4 genomic clones were also transfected with MR. Luciferase activity was measured in the cells transfected with the Kcnn4 genomic clones with (**hatched bars**) and without (**grey bars**) MR transfection. The luciferase activity in the cells transfected with the Kcnn4 genomic clones and MR was also measured in the presence of 10 nM aldosterone (**black bars**). For clarity, luciferase activity measured in the presence of a plasmid, MR and aldosterone are shown in cells transfected with pGL-4.23, clones-3, -5 and -6. Results presented represent mean ± SE from 3 different experiments. *p<0.05– compared to pGL4.23.

The regions which showed enrichment in the ChIP assay were sub-cloned into the pGL4.23 for functional assays. Therefore, clones 1, 3, 5, 6, 7 and 8 were selected based on the results presented in Figure 1C. Clones 1 and 3 are both contained within intron 1. Clone 5 contains a portion of intron 1, an exon 2 and a portion of intron 2 (see supplemental information). Clone 6 is contained within intron 2. Furthermore, both regions 2 and 4 were not obtained (see supplemental information). This was most likely because of the extensive dinucleotide repeats, finding that the regions are not reported as being sequenceable, and lastly, that they are made up of an indeterminate sequence length in the Rat Nov. 2004, Baylor 3.4/rn4 assembly. However, these clones were tested to determine their ability to enhance the expression of luciferase activity following MR-transfection and aldosterone treatment. Clones 1, 5 and 6 all exhibited statistically significant luciferase activity, and the activities were aldosterone and MR dependent (Figure 2). Neither MR not aldosterone alone was able to enhance luciferase activity in clones 1, 5 and 6. Clones 3, 7 and 8 were all exhibited basal luciferase activity only within the presence of MR and aldosterone (Figure 2). All further studies used clones 1, 5 and 6 to characterize the Kcnn4 enhancer activity.

**Figure 3:**
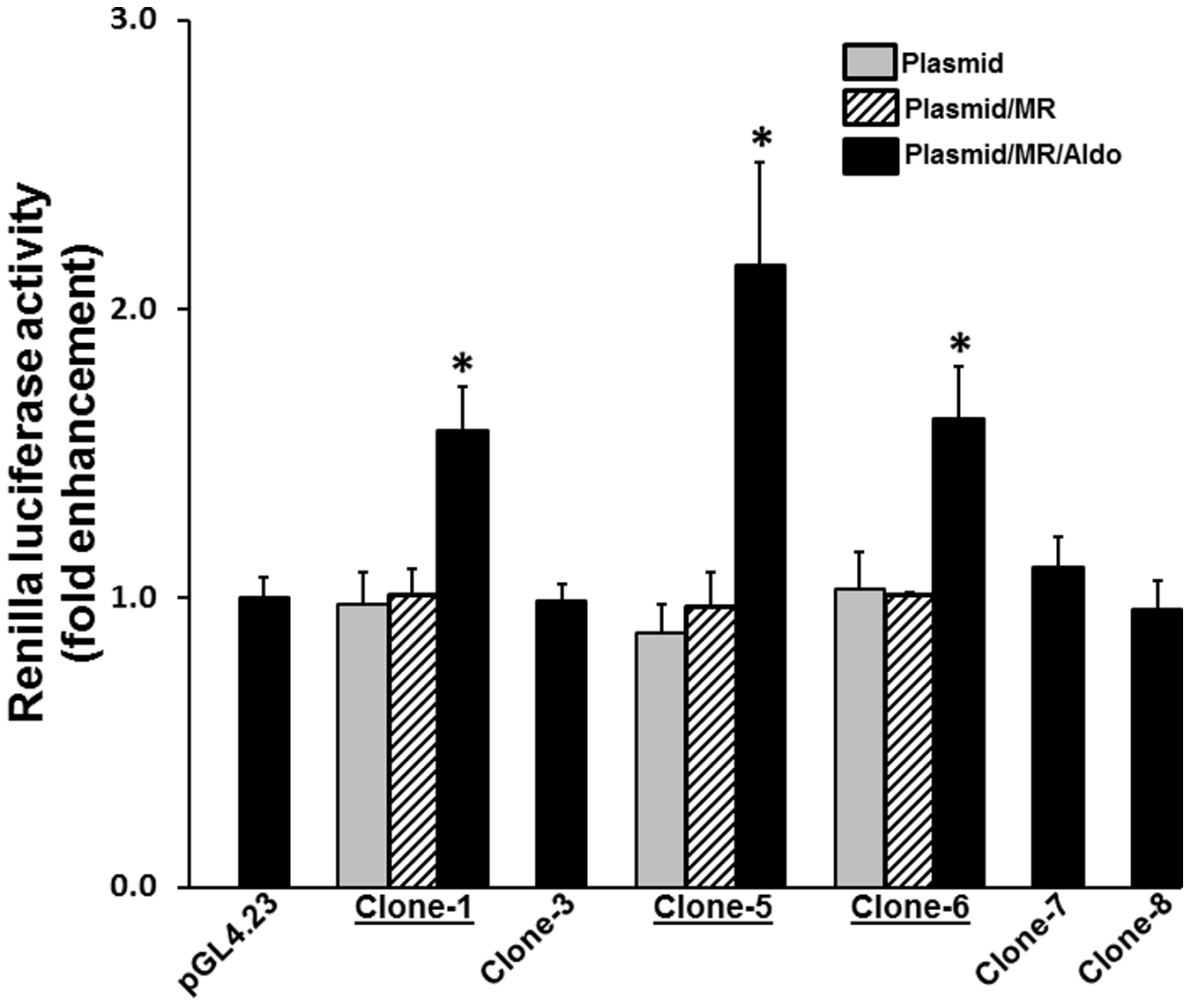
Effect of aldosterone and cortisol concentrations on luciferase activity in mineralocorticoid receptor (MR) and Kcnn4 genomic clones (clone-1, -5 and -6) transfected HEK293T cells. HEK293T cells were transfected with either MR and a vector (pGL4.23) or MR and the respective Kcnn4 genomic clone (clone-1, clone-5 or clone-6). Increasing concentrations (0–100 nM) of either aldosterone or cortisol were determined on luciferase activity. Clone numbers (1, 5 and 6) are indicated to the right. The pGL4.23 transfected cells were unresponsive to both hormones up to 100 nM (data not shown). Murine Mammary Tumor Virus (MMTV) transfected cells exhibited greater than a 30-fold increase in luciferase activity for both hormones with an EC_50_ of approximately 1 nM (data not shown). Fitted curves shown were obtained using Graphpad Prism 4 regression analysis software.

The effects of the mineralocorticoid (aldosterone) and glucocorticoid (cortisol) hormone concentration were examined on clones 1, 5 and 6. In addition, increasing the aldosterone concentration exhibited an enhanced luciferase activity in clones 1, 5 and 6. The increased luciferase activity reached a maximum activity level at the aldosterone concentration of 1–10 nM (Figure 3). In contrast, these clones were unresponsive to cortisol up to 100 nM (Figure 3). These observations are similar to those reported for aldosterone in other systems [23], [24] and indicate that the Kcnn4 genomic clones respond to aldosterone in a physiologically relevant way. These observations also establish that the colonic Kcnn4 gene is a mineralocorticoid, but not a glucocorticoid regulated gene.

**Figure 4:**
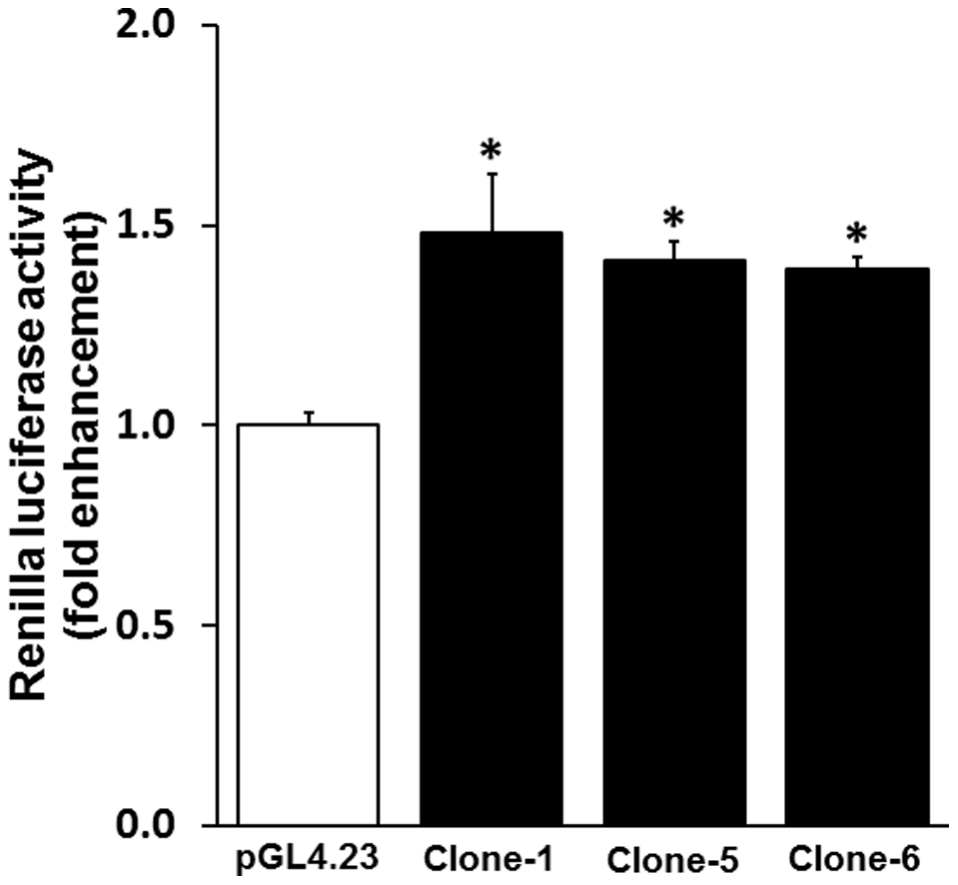
Functional activity of clone-1, clone-5 and clone-6 expressed in CaCo2 cells. The CaCo-2 cells were transfected with either a vector (pGL4.23), clone-1, clone-5, or clone-6. After the transfection, the luciferase activity was measured in the presence and absence of aldosterone (10 nM) treatment. Relative luciferase activity was normalized to the luciferase activity present in the vector transfected cell (pGL4.23; considered 1.0). Data presented represent mean ± SE, from triplicate assays from 3 different experiments). *p<0.05– compared to pGL4.23.

In order to establish that the Kcnn4 gene is also functional in human, the functional activities of clones 1, 5 and 6 were also examined in a CaCo2 cell line. The CaCo2 is a human epithelial colorectal adenocarcinoma cell line which more closely represents the colon. The CaCo2 cells are shown to express MR and the aldosterone responsive function [25]. As shown in Figure 4, aldosterone significantly enhanced the luciferase activity in the CaCo2 cells that transfected with clones 1, 5 and 6. In contrast, the aldosterone did not activate the luciferase activity in vector transfected CaCo2 cells (Figure 4). This observation establishes that the Kcnn4 genomic regions present in clones 1, 5 and 6 was involved in the aldosterone dependent regulation of Kcnn4 activity in the human colon.

**Figure 5:**
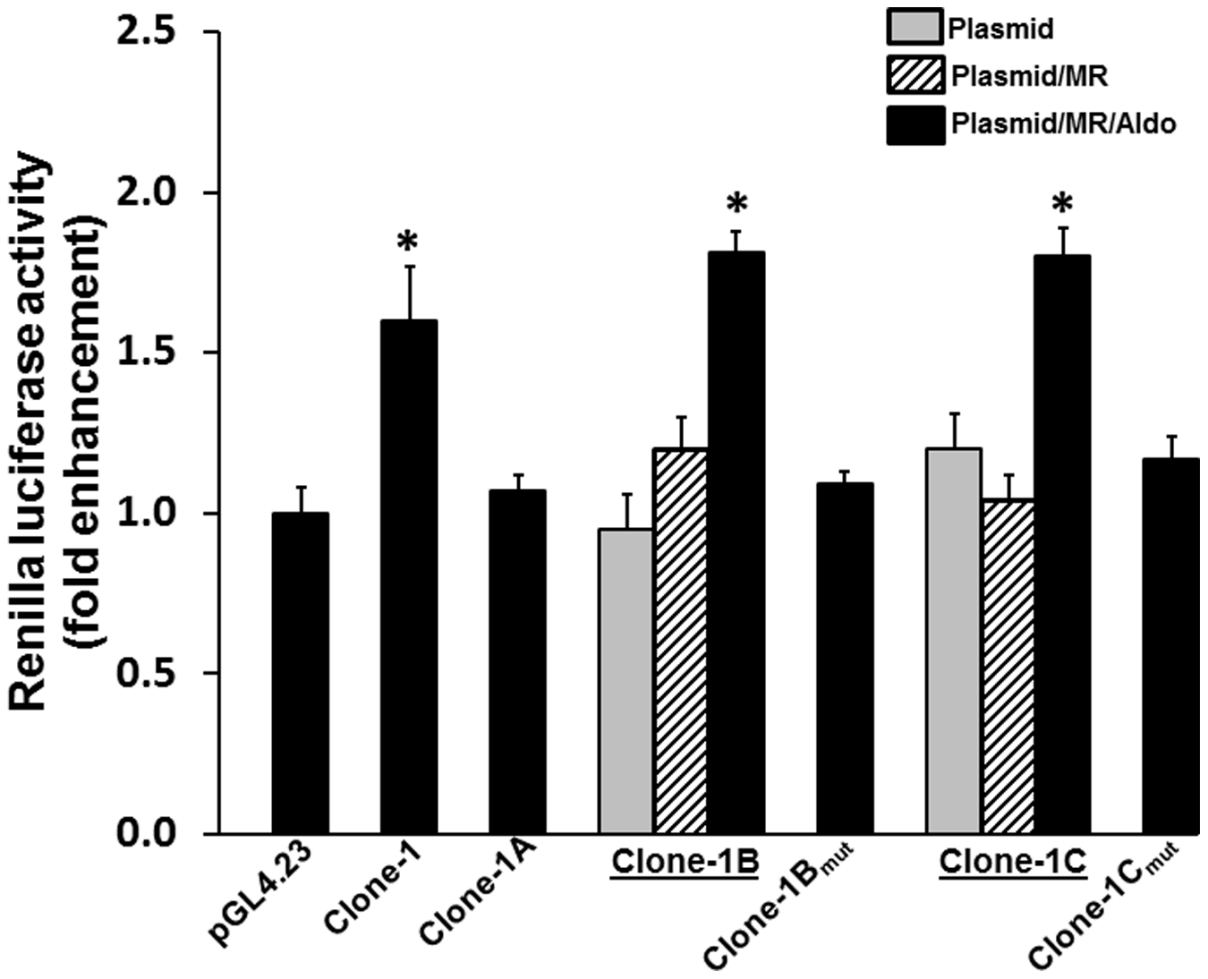
Sub-localization of mineralocorticoid hormone responsive element (MRE) in clone 1. Sub-clones of clone-1 were made and tested for MR/aldosterone responsiveness in HEK 293T cells. Clone-1 was sub-cloned into thirds, clone-1A, clone-1B and clone-1C representing the left, middle and right thirds, respectively. Clone-1B and clone-1C exhibited enhanced luciferase activity. Putative MREs were identified in clone-1B and clone-1C using TESS and MatInspector bioinformatics tools. The sequence TCTTGA GTGTGTTCT in clone-1B (candidate MRE underlined) was mutated to TCTTGcGTGcacgac (clone-1B_mut_) (see Figure S1 for sequence). The candidate MRE GGCTCT GCGTGTTCT in clone-1C was mutated to GGCTgTGCGcacgac (clone-1C_mut_). The absence of the enhanced luciferase activity in the HEK293T cells transfected with clone-1B_mut_ and clone-1C_mut_ showed a loss of function, thus demonstrating functionality of the MREs localized in clone-1B and clone-1C. Data presented represent mean ± SE of triplicate assays from 3 different experiments. *p<0.05– compared to pGL4.23.

Studies were designed to sub-localize the MR-responsive activity to assist in the final identification of the responsive elements. The clone-1, -5 and -6 contain inserts of 1886, 1336 and 1558 bp, respectively. Clones 1, 5 and 6 were sub-cloned into thirds and designated respectively as A, B and C, with each one representing the left, middle and right thirds. The sub-clones (and succeeding sub-clones) were made with overlaps to avoid an inadvertent disruption of the MREs (see supplemental information). As shown in Figure 5, the sub-localized enhancing affects both the middle (clone-1B) and last third (clone-1C) of clone-1. The clone-1B and -1C were analyzed using the Transcription Element Search System (TESS) (URL: http://www.cbil.upenn.edu/tess) and MatInspector [26] to identify potential HREs. The putative MRE1 (GGCTCTgcgTGTTCT) and MRE2 (TCTTGAgtgTGTTCT) specific sequences are identified to localize in clone-1C and clone-1B, respectively. To prove that indeed the MR binds to the putative MRE, MRE1 (GGCTCT GCGTGTTCT ) was mutated to GGCTgTGCGcacgac (clone-1B_mut_), while MRE2 (TCTTGA GTGTGTTCT ) was mutated to TCTTGcGTGcacgac (clone-1C_mut_) (see Figure S1 for sequence). These changes primarily alter the sequence TGTTCT, and are by far the most conserved portion of the response element, and would thus be expected to destroy the response activity. The mutation of these regions abrogates the activity (Figure 5; clone-1B_mut_ and clone-1C_mut_), and thus implicates these as the MR response elements.

**Figure 6:**
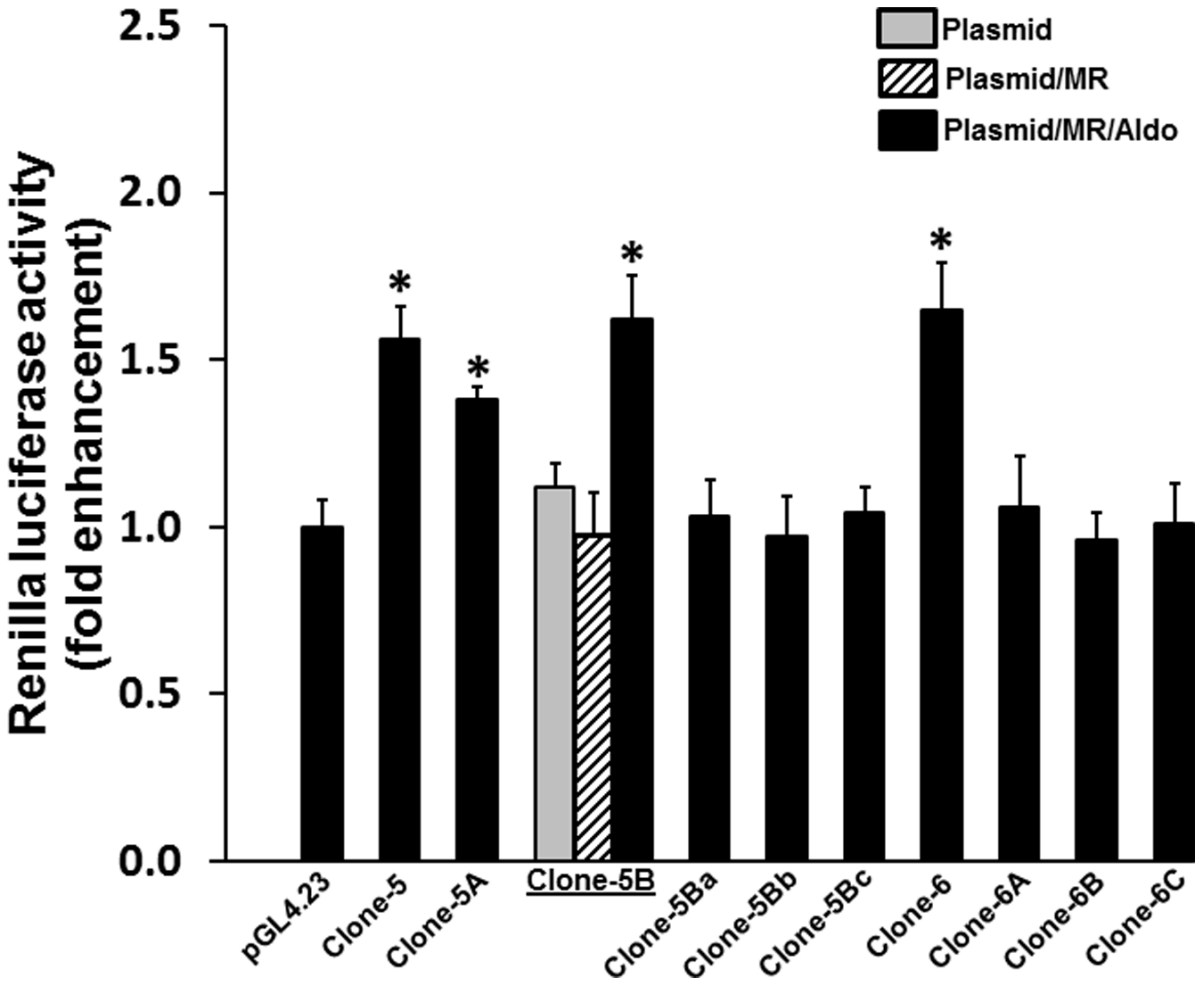
Sub-localization of mineralocorticoid response elements (MRE) in clone-5 and clone-6. The clone-5 was sub-cloned into thirds, clone-5A, clone-5B and clone-5C representing the left, middle and right thirds of clone-5, respectively. Clone-5A, -5B and -5C were tested for MRE/aldosterone responsiveness in the HEK293T cells. The HEK293T cells transfected with clone-5A and clone-5B exhibited a significantly higher luciferase activity compared to the pGL4.23 vector transfected cells. Neither clone-5A nor clone-5B contained candidate MREs as analyzed using the Transcription Element Search System (TESS) or MatInspector. Further sub-clones of clone-5B were made (clone-5Ba and clone-5Bb that represent the left and right half of clone-5B, respectively). (See Figure S1 for the sequences). Luciferase activity was not significantly altered in the HEK239T cells transfected with clone-5Ba and clone-5Bb, compared to the cells transfected with a vector (pGL4.23). Clone-6 was also sub-cloned into thirds (clone-6A, clone-6B, and clone-6C). Similar to the clone-5Ba and clone-5Bb transfected cells, luciferase activity was also not significantly altered in the HECK293T cells transfected with clone-6A, clone-6B and clone-6C compared that of cells transfected with a vector (pGL4.23). Data presented represent mean ± SE of triplicate assays of 3 different experiments. *p<0.05– compared to pGL4.23.

The studies were also performed to sub-localize the MR-responsive activity in clone-5 and clone-6. Similar to clone-1, clone-5 (5A, 5B and 5C) and clone-6 (6A, 6B and 6C) were also sub-cloned into thirds. As shown in Figure 6, clone-5A was partially active, while clone-5B exhibited somewhat more activity. Additionally, the TESS, MatInspector analyses and visual inspection all revealed the absences of a good HRE consensus sequence in clone-5B and clone-5C. In an attempt to functionally localize the response elements, two sub-clones of clone-5B, clone-5Ba and clone-5Bb representing the left and right halves of 5B with a 21 bp overlap, respectively, (Figure S1) were tested for luciferase activity. Both clone-5Ba and clone-5Bb were found to be unresponsive to the aldosterone/MR (Figure 6). The absence of luciferase activity in clone-5Ba and clone-5Bb might be due to the separation of the cooperative elements upon sub-cloning. Similar to clone-5B, sub-clones of clone-6 (clone-6A, clone-6B and clone-6C) were also losing the luciferase enhancer activity. Lastly, the sub-clones of clone-6 were overlapped by 50 bp.

## Discussion

The rat Kcnn4 gene is responsive to aldosterone, as demonstrated by the association of the elevated serum aldosterone in the rats fed a Na-free diet with an elevated Kcnn4 mRNA in the colonic mucosa, and by the ability of aldosterone to increase Kcnn4 transcription in vitro in the isolated colonic mucosa [9]. In this study, we demonstrated this through the direct MR action on the gene. The regions surrounding the gene were examined for MR binding using ChIP assays. Classically, interactions between the proteins and its counterpart nucleotide binding elements were determined using classical methods such as electrophoretic mobility shift assays followed by super-shift assays and using the antibodies to show the specificity of the binding protein. However, this technique suffers from major limitations such as in vitro complex formation and lack of functional information followed by binding [27]. Hence, we chose to use an alternate method to identify the MRE by using chromatin immunoprecipitation, which specifically precipitated the protein-nucleotide complex formed in vivo under physiological conditions followed by functional assays using a luciferase gene expression. Finally, the involvement of the MRE binding was confirmed by site directed mutagenesis of the nucleotides in the putative MRE binding site followed by a functional assay of the mutants.

While no obvious aldosterone binding was found in the promoter region, binding was found in a broad region spanning parts of introns 1 and 2. In contrast, aldosterone binding was not detected in these regions within normal rats where the aldosterone levels are too low. This correlation between high levels of aldosterone and MR binding to the gene lends support to the concept that an increased expression of Kcnn4 is a direct result of the MR activity. The functional assays then confirmed the presence of enhancers in three clones in response to MR transfection and aldosterone treatment. In clone 1, two regions showed enhancer activity and contained MREs recognized by computational means. Both regions lost their activity upon mutation, demonstrating their functionality. MRE1 was not conserved in a mouse or man, while MRE2 was fully conserved in a mouse but not in a man (not shown). While interspecies conservation has been frequently referenced as a good predictor of HRE functionality [13], others have found that conservation of certain regulatory sites was low [28], [29].

Steroid hormone response elements usually consist of palindromic hexameric repeats separated by 3 bp [11], [12], [13], [14], [30]. Neither half site displayed close homology to the consensus (AGAACAnnnTGTTCT), while the “left” in particular can show extremely wide variation [13], [14]. Therefore, MRE1 falls within the description of an HRE palindromic repeat (GGCTCTgcgTGTTCT; consensus bases underlined: the underlined G and C in the left repeat are the most highly conserved [13], [14]. Furthermore, MRE2 (TCTTGAgtgTGTTCT) shows a wide variation from the consensus (in the left site). Thus, MRE2 in particular, and also MRE1, may consist only of half sites or else of repeats with the left site showing a high variation from the consensus. The sites not bioinformatically identified in clones 5 and 6 will of course show a substantial variation from the consensus. The generally low homology to the overall consensus sequence tallies with their modest enhancement activity, consistent with the results of others and showing that the mutants of the consensus sequence show reduced transcription activation and a reduced hormone binding for ER [31].

It is generally accepted that the majority of HREs are located distally to their promoters and can be either upstream or downstream. Glucocorticoid response elements are distributed equally upstream and downstream of the TSS of responsive genes with many elements being >10 kb from the TSS [13]. Similar results have been found for estrogen response elements (EREs) [32], [33] and androgen response elements (AREs) [34]. We are not aware of any similar studies on the MREs. Furthermore, while functional studies have revealed a maximum of two intronic HREs for PR [35] and GR [36], we are not aware of any functional studies identifying any intronic MREs. Our results are therefore the first to demonstrate intronic MREs. In addition to the two MREs identified, three other regions were identified which are modestly MR and aldosterone-responsive, but which became inactive upon further sub-cloning. The loss of activity upon sub-cloning suggests a cooperativity among the components of a clone, perhaps comprising multiple HREs, half sites, or transcription factor-binding sites, as others have in different systems [37]. [30]. We suggest that our findings of up to five response elements in Kcnn4 may represent a transcriptional control mechanism typified by multiple response elements with modest activity individually. These could easily be overlooked because of their modest activity, and more so if the interactive components are separated during cloning. Furthermore, our finding of response elements in multiple introns is novel as it has not been seen with PR, GR, or ER. The clones 1, 5 and 6 were responsive to aldosterone, but not to cortisol, a glucocorticoid hormones (Figure 2). These hormones act in many situations as equivalently effective ligands for MR [38]. While target tissue specificity is conferred on the epithelial tissues by the enzyme 11β-hydroxysteroid dehydrogenase type 2, which converts cortisol to an inactive cortisone [29], [39], this cannot be said of the HEK293T cells (which are devoid of the enzyme [40], and in which cortisol is fully active on MMTV. Others have suggested that a given HRE DNA sequence can have distinct effects on the receptor function, perhaps through allosteric effects on the receptor in turn altering co-regulators interacting with the receptor [41], [42]. This may explain the insensitivity of the clones to cortisol. Consistent with the insensitivity to cortisol, others have found that the GR-specific agonist RU 28362 activates electroneutral Na-Cl absorption in the rat distal colon (demonstrating its activity in the tissue), but it has no effect on the K^+^ transport [43].

## Supporting Information

Figure S1 **Sequence of Clones 1, 5, and 6 and of selected sub-clones.** Clones 5 and 6 overlap by 138 bp, all contiguous sub-clone inserts overlap by approximately 50 bp, and 5Ba and 5Bb overlap by 21 bp. Potential HREs are underlined. Exon 2 (99 bp) in 5B is underlined.
(DOCX)

Table S1 **Primer sets used in ChIP.** Primer sets are numbered with reference to the genomic DNA block they are directed to amplify. For example, Primer set 1 (forward and reverse primers) RT-PCR amplify approximately 100 bp in DNA block 1, approximately 20 kb upstream of the presumed Kcnn4 transcription start site (TSS).
(DOCX)

Table S2 **Primer sets used for cloning candidate enhancer regions from genomic DNA.**
(DOCX)
