# Supplementary material for: Multiple Mineralocorticoid Response Elements Localized in Different Introns Regulate Intermediate Conductance K+ (Kcnn4) Channel Expression in the Rat Distal Colon
Source: PLoS One. 2014 Jun 5;9(6):e98695. doi: 10.1371/journal.pone.0098695 (PMC4047071; doi:10.1371/journal.pone.0098695)
Supplement: Figure S1 — Sequence of Clones 1, 5, and 6 and of selected sub-clones. Clones 5 and 6 overlap by 138 bp, all contiguous sub-clone inserts overlap by approximately 50 bp, and 5Ba and 5Bb overlap by 21 bp. Potential HREs are underlined. Exon 2 (99 bp) in 5B is underlined. (DOCX) [file pone.0098695.s001.docx]

**Figure - S1: Sequence of Clones 1, 5, and 6 and of selected sub-clones.** Clones 5 and 6 overlap by 138 bp, all contiguous sub-clone inserts overlap by approximately 50 bp, and 5Ba and 5Bb overlap by 21 bp. Potential HREs are underlined. Exon 2 (99 bp) in 5B is underlined.

**Clone-1**:

GGAGGGAGGCTGGTGTTTTATACCTCTCACTGTGAAGGAGGAGGGCTGGGGTCTGCACCGCGGCTGTGTGAGAGAGGATGCTGGCAACTTACACCCTTGAGAGTGAAGGAGAGAGGAGATCTGGGAGTCTTATACCCTGGGTATGAAAGAGGAGGACTGGGCTTTGCACTCCTGGGAGTGAGGAAGGAGGGCTGGGGTTTGCACCCCTGGCTGTGAGAGAAGAGAGCTGGTCTGGACTCTCGGGTCTAAGGGAGGGGATCTGAAGTCTTACACTTCTTAAGATCTAAGGGAAGAGGGCTGGGTCTGGATCCCTTGGTGTGGGAGAAGCAGATGGGGGTTCTGCACCCCTGAAACCTGAGTTACTGTGAATCCATCCTACTCCTTCCCTCCCTCCCCCACGCACAGAAGGGAGACAGTTCTCAGTGTTAGCCTTTGGGTGTTATCAGACCACTGGGGGTCTGGGCCATGTTGAGGCCCATGCAAGGCTGACCCTTGATAGCCCATCTTTGGTGAAGGGAGGGCGAGGAAGAAAGACTTTGTCATTAGGCGTCTCCTTGCTCCTTATCTAGCCTCTGCCATTTACCCAGTGCCACCAGGCTGTGCCCTGAAGTAACCGACAGGGAGAAGGGTGGGGAGTGGAGCCTCCACCCCACCTCTAACATACAGGATGCCGTTACCCCCAGAAGACACCTGTTATTCATTTACCAGATAGTGGTTTACCATGGCTTGAACTCCCTTGTCCCTAGGGGAGAGGAGACTTGGGGTTCAGACTTGAGTCTGCAGGAGGAGTCTCAAAAAGTCTCACCAGGCATGGAATCCAAGCACTGGGGAAGTGGGAGGCAAGAGAATCAGAAGGTTAGGCCCAGCCTATGCTACATACTGAGTTGGAGGCCAGCCTGGGCTACACAAGACCTTATCTCAAAAAACAAAAATAATTCCAAACAACGGGGTGGGGGA**AGAACA**CAC**TCAAGA**GAGCTGTCAATCAATCTCTGGAAGACCAGGGAGTGAGGTGGGGCTGGGACAGGTCTTAGCAAGCCCCGGGAATATTCTGGCTCTGCTCAGGTTTGTGGCATAAGAGAGATGGAGGGCAAAGGTTGGGTCTGCGGTCTGAAGGGGAGACGCACTTGTTCAACAGAAAAGTTGAGGCAGGGGCTGGGCATGTGTGCGCACGCGCGTGTACTTAGGTGGACCGTTCTTTGGGAGAACTTCTGGGCCCTGGGATTGAGGATTAGGAAGGGGCAGCAGCTGCTGTGAAGTGACCGGCAGCCACATGGACGGTCAGGGCAGGACAGCTTCCTTCAGCCAAGTGGCCAAGGAGAGGGCAGCCACTGCAAGGGGGTGGGGCTGTCCAGAGCCATGCCAACAGGAAACATGGCTGGGGCCGGCCCCGGCGCTCCCACGCTCCTGCACCGCGCCCAGCAGGGGAGGTGGGGGACCTGGGAGTCCAGGCAGAAGGGAAGGGACCAGGGGGTCTCTTTTCCTTCATCTCTCCCTTTTCACTACCTACCCAGGCCCTTTCCTCCAACTGTGATTCTGAAGGAGCAGATGCCACGCTCTGAGCCTCAGTTTTCCCATCTCTGCAACCGTAGCCATGAGTGCTCTTCCAATCTTTGTTTGAGACAAGGTAGCCCAGGTTGGCCTGGAACTCGCTATGTAGCTGAGGATGACCGTGTACTTCTGATCCTCCTGCCGCCGCTTCCCCAAGTGCAGGGACCACAGGCAGGTACCACAACACTCGGTTTACACAGGATGAGGGTCAAACGAA**GGCTCT**GCG**TGTTCT**AGGTAAACACTCTTCCAACTGAGCTACATCGACACCCTAAAACCCCCTCCTATGGCTGCCTTGGTGACCTCCATGCCCCCTCCCTCCGCAACTCCATCTCAATCGA

**Clone 1A**:

GGAGGGAGGCTGGTGTTTTATACCTCTCACTGTGAAGGAGGAGGGCTGGGGTCTGCACCGCGGCTGTGTGAGAGAGGATGCTGGCAACTTACACCCTTGAGAGTGAAGGAGAGAGGAGATCTGGGAGTCTTATACCCTGGGTATGAAAGAGGAGGACTGGGCTTTGCACTCCTGGGAGTGAGGAAGGAGGGCTGGGGTTTGCACCCCTGGCTGTGAGAGAAGAGAGCTGGTCTGGACTCTCGGGTCTAAGGGAGGGGATCTGAAGTCTTACACTTCTTAAGATCTAAGGGAAGAGGGCTGGGTCTGGATCCCTTGGTGTGGGAGAAGCAGATGGGGGTTCTGCACCCCTGAAACCTGAGTTACTGTGAATCCATCCTACTCCTTCCCTCCCTCCCCCACGCACAGAAGGGAGACAGTTCTCAGTGTTAGCCTTTGGGTGTTATCAGACCACTGGGGGTCTGGGCCATGTTGAGGCCCATGCAAGGCTGACCCTTGATAGCCCATCTTTGGTGAAGGGAGGGCGAGGAAGAAAGACTTTGTCATTAGGCGTCTCCTTGCTCCTTATCTAGCCTCTGCCATTTACCCAGTGCCACCAGGCTGTGCCCTGAAGTAACCGACAGGGAGAAGGGTGGGGAGTGGAGCCTCCACCCCACCTCTAACATACAGGATGCCGTTACCCCCAGAAGAC

**Clone 1B**:

GTCCATGTGGCTGCCGGTCACTTCACAGCAGCTGCTGCCCCTTCCTAATCCTCAATCCCAGGGCCCAGAAGTTCTCCCAAAGAACGGTCCACCTAAGTACACGCGCGTGCGCACACATGCCCAGCCCCTGCCTCAACTTTTCTGTTGAACAAGTGCGTCTCCCCTTCAGACCGCAGACCCAACCTTTGCCCTCCATCTCTCTTATGCCACAAACCTGAGCAGAGCCAGAATATTCCCGGGGCTTGCTAAGACCTGTCCCAGCCCCACCTCACTCCCTGGTCTTCCAGAGATTGATTGACAGCTC**TCTTGA**GTG**TGTTCT**TCCCCCACCCCGTTGTTTGGAATTATTTTTGTTTTTTGAGATAAGGTCTTGTGTAGCCCAGGCTGGCCTCCAACTCAGTATGTAGCATAGGCTGGGCCTAACCTTCTGATTCTCTTGCCTCCCACTTCCCCAGTGCTTGGATTCCATGCCTGGTGAGACTTTTTGAGACTCCTCCTGCAGACTCAAGTCTGAACCCCAAGTCTCCTCTCCCCTAGGGACAAGGGAGTTCAAGCCATGGTAAACCACTATCTGGTAAATGAATAACAGGTGTCTTCTGGGGGTAACGGCATCCTGTATGTTAGAGGTGGGGTGGAGG

**Clone 1C**:

ATTAGGAAGGGGCAGCAGCTGCTGTGAAGTGACCGGCAGCCACATGGACGGTCAGGGCAGGACAGCTTCCTTCAGCCAAGTGGCCAAGGAGAGGGCAGCCACTGCAAGGGGGTGGGGCTGTCCAGAGCCATGCCAACAGGAAACATGGCTGGGGCCGGCCCCGGCGCTCCCACGCTCCTGCACCGCGCCCAGCAGGGGAGGTGGGGGACCTGGGAGTCCAGGCAGAAGGGAAGGGACCAGGGGGTCTCTTTTCCTTCATCTCTCCCTTTTCACTACCTACCCAGGCCCTTTCCTCCAACTGTGATTCTGAAGGAGCAGATGCCACGCTCTGAGCCTCAGTTTTCCCATCTCTGCAACCGTAGCCATGAGTGCTCTTCCAATCTTTGTTTGAGACAAGGTAGCCCAGGTTGGCCTGGAACTCGCTATGTAGCTGAGGATGACCGTGTACTTCTGATCCTCCTGCCGCCGCTTCCCCAAGTGCAGGGACCACAGGCAGGTACCACAACACTCGGTTTACACAGGATGAGGGTCAAACGAA**GGCTCT**GCG**TGTTCT**AGGTAAACACTCTTCCAACTGAGCTACATCGACACCCTAAAACCCCCTCCTATGGCTGCCTTGGTGACCTCCATGCCCCCTCCCTCCGCAACTCCATCTCAATCGA

**“Clone 2” (untested)**:

CCCTCCGCAACTCCATCTCAATCGAACAAACTTGTTTTTCTTTTTCTTTTTTTTCCTTTTTCTTTCTTTTTTTTTTTTTTTTTCCGGAGCTGGGGACTGAACCCAGGGGCTTGCGCTTACTAGGCAAGCGCTCTACCACTGAGCTAAATCCCCAACCCCAAACTTGTTTTTCACCAAACAAAGACACCTGCTCCTCAGGAGGGGCGGGTGCCAAGAATGTCGGAAGGANNNNNNNNNNNNNNNNNNNNNNNNNNNNNNNNNNNNNNNNNNNNNNNNNNACACAA

CCACACATACACACACCACACACACACCACACATACACACACCACACACACCACACACACACATGCACACCACACATACACACACTACACATACACGTACACGGCACATGCACACACACACATATGACACACACACCGCACACGCACACACACACACATGCACACCACACATACACATACCACACACACACACATGCACACCACACATACACATACCACACATACACATGCACACCACACATACACATACCACACACACACACACATGCACACCACACAT

ACACACACCACACACACACATGCACACCACACATACACACACCACACACACCGCACACACACACAATGCACACCACACATACACACACCACACACACCGCACACACACACATGCACACCACACACACACACACACACACACACACACACACACACACACACACCAGTTGGGCAGTAGCTCAGTTGGTAGAGTACTTGCTTAGCATGCGCAAAGGCCCTGGGCTTGATTTCCTGCTCTGGACTGTGAGGCTGCTTGCCTGCAATCCTAGCATTTAGTAGGTCTAGAAGCAGATGGAACTCTCAGAGAGAGGGGCATAAGAGACCCCCGTCTGGGTGTAGAATTGGAAAAGGGGACCCCCTACCCAACCCCCGCCGTAGGAGAAATGAATTGCATTGTGGGAAAGTGGCTTGTGTCTGAAGCGATAGGGGCTGAGACAAACTG

**“Clone” 4 (untested)**:

CTGCTCTGGACTGTGAGGCTGCTTGCCTGCAATCCTAGCATTTAGTAGGTCTAGAAGCAGATGGAACTCTCAGAGAGAGGGGCATAAGAGACCCCCGTCTGGGTGTAGAATTGGAAAAGGGGACCCCCTACCCAACCCCCGCCGTAGGAGAAATGAATTGCATTGTGGGAAAGTGGCTTGTGTCTGAAGCGATAGGGGCTGAGACAAACTGAAAAACTGAACGGGGAACTGGGGAAGGGTGAAGGATGACGGGGGAGGGGAAGGCTGGGGACAACAGCGCCATTGTTCAGACAGGGGTGGGGGGAGGCCCCCCGGGGGCAGGAAACTGGCCCTCTTCCCCTGCTCAGAGGCGGGGTGCTGGCGTGGGCCCAGCCAGGGAAACTGGCAGAGGCTTCTGAGGAACAGGTGCAGGGTTCAATGAGGGAACCGGGGCAAGCCCTGCCCTGGGACAGTATCTCCCTATGGGCATCGGCTCATCTTAGGGAACTCAGCAATTCTCACCCCCCAACTCTCTAAATCCTTCCTCCCTAAATATTAGCACTCTCCAAGGCATTGCCACACAAAGAGAGGAAGAGAAAAAAACTTCTGGTACTTTATGTAGGCCCTGAACTTCCGCCCCATGTGCCCTGGGCACATAGCCAGAGGAAGTGGTAGGAGAGGNNNNNNNNNNNNNNNNNNNNNNNNNNNNNNNNNNNNNNNNNNNNNNNNNNNNNNNNNNNNNNNNNNNNNNNNNNNNNNNNNNNNNNNNNNNNNNNNNNNNNNNNNNNNNNNNNNNNNNNNNNNNNNNNNNNNNNNNNNNNNNNNNNNNNNNNNNNNNNNNNNNNNNNNNNNNNNNNNNNNNNNNNNNNNNNNNNNNNNNNNNNNNNNNNNNNNNNNNNNNNNNNNNNNN

NNNNNNNNNNNNNNNNNNNNNNNNNNNNNNGGAGCTGGGGCTGGGGGTCTTGGGGTTCATTCCCCAGATTAGAGAAGGGCAGAATGATGCTCTAGGAGACTTGAGTGACTTGAGTGACAGTCTCTAGTCTTTTCTGGAATTGGGTTCGGGGCAGGCCCAGGAGATGGAAGGAGCATGCTCGTGAGGCTGGATGTCTTTTTCTAGACATCACATGCATGCACAAATGAGGAACTATTTTTCATAGGGAAAGAGAAAGAATGTGAGTTTCAAGGCTCCCTGATGGTAGGGGAGTGGAGGGAAGTGAGGATGAGACATGGCTGTCTGGCTTAGATGATCTCCATCTGCCATAACCCAAGTAGACTC

**Clone 5**:

GGGCAGAATGATGCTCTAGGAGACTTGAGTGACTTGAGTGACAGTCTCTAGTCTTTTCTGGAATTGGGTTCGGGGCAGGCCCAGGAGATGGAAGGAGCATGCTCGTGAGGCTGGATGTCTTTTTCTAGACATCACATGCATGCACAAATGAGGAACTATTTTTCATAGGGAAAGAGAAAGAATGTGAGTTTCAAGGCTCCCTGATGGTAGGGGAGTGGAGGGAAGTGAGGATGAGACATGGCTGTCTGGCTTAGATGATCTCCATCTGCCATAACCCAAGTAGACTCCAAATTGGAAATCCTCCTGTCTCAGCCTACTGAGGGCTGGAATCATACGTGTGACCTACACACTAGACTCCAGAATGGTTTGAGATGTCAGGAGGGCCTGGTTCGGGAGCCAGATGGTTGGGAGTGCAGAGATGGCCTTTCTGAAGGAAGGGAATGGGGGTGGGTGGAGTGGGAGCGCCACTCACTGTCTTTTGTTGCCCACAGTGGGTGCTGTACCTGCTCTTGGTTAAGTGTTTAATCACGCTGTCCACTGCCTTCCTCCTTTGTCTTATTGTGGTCTTCCATGCCAAGGAGGTCCAGGTAAGCTGGGTCCCACCCCTCAGCTTTGCTCTACGTG

TACCCTTCTCCTTCCCTGAGATGCATGAGTCTACACTTTTGCTTGCTGGGGTGGAACCAGCCACAGACAGACAGACAGACAGACAGATTCTGCTGGCTTAGCTGATTCATCCTCCAGTGTAGCCCCCATGTGTTTCCTTGCCACTTAGAGATGGTGGCAACGCCTGCCCTTCCAGCTCCCAAGTCCCAAGACCATTTACTTTTTTAAAATTAATTTCGGTGCTGGGGACTGAAATTTAGGACCTTGCACTGAGCCACGCCCCCAGCCCCTCACTGGGGGATTCTAGGCAGGGGATTTTTTTTTCCCTCTTTTTTTCAGAGCTGGGGACCGAACCCCAGGGCCTTGTGCTCGCTAGGCAAGCGCTTTACTGCTGAGCTAAATCCCCAACCCCGTGTACTTAAATTTTTAACATTTATTAATTTATGGGGCACACTTAGAAGTCAGAAGGCAAATTATTCAAGTCAGTTCTCTCCTTCTCCCACGTGGGGTTTTTAGGAATCAAACTCAGGTCATAGTGTTTGGAGGTAAAAGCCTTGGCTCACTGAGCCATCGCATAGGCCCAAAGAATGTGAATTTTAACTGAGCGCCTCTTAGGTGCCCAGCACATCCCAGGGTATTGCTGTACATGGCTGAGAATACTGTCTTACGTGGGAGTCAGGCAACTGGGAAAAAGATCTGGAGAAATCAAAATGGGAATGGCATTGTGTTTCAG

**Clone 5A**:

GGGCAGAATGATGCTCTAGGAGACTTGAGTGACTTGAGTGACAGTCTCTAGTCTTTTCTGGAATTGGGTTCGGGGCAGGCCCAGGAGATGGAAGGAGCATGCTCGTGAGGCTGGATGTCTTTTTCTAGACATCACATGCATGCACAAATGAGGAACTATTTTTCATAGGGAAAGAGAAAGAATGTGAGTTTCAAGGCTCCCTGATGGTAGGGGAGTGGAGGGAAGTGAGGATGAGACATGGCTGTCTGGCTTAGATGATCTCCATCTGCCATAACCCAAGTAGACTCCAAATTGGAAATCCTCCTGTCTCAGCCTACTGAGGGCTGGAATCATACGTGTGACCTACACACTAGACTCCAGAATGGTTTGAGATGTCAGGAGGGCCTGGTTCGGGAGCCAGATGGTTGGGAGTGCAGAGATGGCCTTTCTGAAGGAAGGGAATGGGGGTGGGTGGAGTGGGAGCGCCACTCACTGT

**Clone 5B**:

CTTTCTGAAGGAAGGGAATGGGGGTGGGTGGAGTGGGAgc**gccact**CAC**TGTCTT**TTGTTGCCCACAGTGGGTGCTGTACCTGCTCTTGGTTAAGTGTTTAATCACGCTGTCCACTGCCTTCCTCCTTTGTCTTATTGTGGTCTTCCATGCCAAGGAGGTCCAGGTAAGCTGGGTCCCACCCCTCAGCTTTGCTCTACGTGTACCCTTCTCCTTCCCTGAGATGCATGAGTCTACACTTTTGCTTGCTGGGGTGGAACCAGCCACAGACAGACAGACAGACAGACAGATTCTGCTGGCTTAGCTGATTCATCCTCCAGTGTAGCCCCCATGTGTTTCCTTGCCACTTAGAGATGGTGGCAACGCCTGCCCTTCCAGCTCCCAAGTCCCAAGACCATTTACTTTTTTAAAATTAATTTCGGTGCTGGGGACTGAAATTTAGGACCTTGCACTGAGCCACGCCCCCAGCCCCTCACTGGGGGATTCTAGGCAGGGGATTTTTTTTTCCCTCTTTTTTTCAGAGCTGGGGACCGAACCCCAGGGCCTTG

**Clone 5Ba**:

CTTTCTGAAGGAAGGGAATGGGGGTGGGTGGAGTGGGAgc**gccact**CAC**TGTCTT**TTGTTGCCCACAGTGGGTGCTGTACCTGCTCTTGGTTAAGTGTTTAATCACGCTGTCCACTGCCTTCCTCCTTTGTCTTATTGTGGTCTTCCATGCCAAGGAGGTCCAGGTAAGCTGGGTCCCACCCCTCAGCTTTGCTCTACGTGTACCCTTCTCCTTCCCTGAGATGCATGAGTCTACACTTTTGCTTGCTGGGGTGGAACCAGCCACAGACAGACAGACAGACAGACAGATTCTGCTGGCTTAGCTGATTCATCCTCC

**Clone 5Bb**:

GGCTTAGCTGATTCATCCTCCAGTGTAGCCCCCATGTGTTTCCTTGCCACTTAGAGATGGTGGCAACGCCTGCCCTTCCAGCTCCCAAGTCCCAAGACCATTTACTTTTTTAAAATTAATTTCGGTGCTGGGGACTGAAATTTAGGACCTTGCACTGAGCCACGCCCCCAGCCCCTCACTGGGGGATTCTAGGCAGGGGATTTTTTTTTCCCTCTTTTTTTCAGAGCTGGGGACCGAACCCCAGGGCCTTG

**Clone 5C**:

GCTGGGGACTGAAATTTAGGACCTTGCACTGAGCCACGCCCCCAGCCCCTCACTGGGGGATTCTAGGCAGGGGATTTTTTTTTCCCTCTTTTTTTCAGAGCTGGGGACCGAACCCCAGGGCCTTGTGCTCGCTAGGCAAGCGCTTTACTGCTGAGCTAAATCCCCAACCCCGTGTACTTAAATTTTTAACATTTATTAATTTATGGGGCACACTTAGAAGTCAGAAGGCAAATTATTCAAGTCAGTTCTCTCCTTCTCCCACGTGGGGTTTTTAGGAATCAAACTCAGGTCATAGTGTTTGGAGGTAAAAGCCTTGGCTCACTGAGCCATCGCATAGGCCCAAAGAATGTGAATTTTAACTGAGCGCCTCTTAGGTGCCCAGCACATCCCAGGGTATTGCTGTACATGGCTGAGAATACTGTCTTACGTGGGAGTCAGGCAACTGGGAAAAAGATCTGGAGAAATCAAAATGGGAATGGCATTGTGTTTCAG

**Clone 6**:

GGTGCGTGTTCATGTATAGTTTGGCCACGAACCAGTGGCGGAATCGGACTTGGTTGAGCGCCCCGATGCTGCGGTAAGACGCGTTGAGCAGGACCCCGCTACGCAGAAGTACCGCGCGAGGCACCAGGTAGAGACGTAGCAGCATGGCCAGGGACAGCAACGCCTCGCCTTCGCCCAGGAAGCCCGGCCAGGCCTGTGAGTCTGTGGCCTCCCCCGCCAGGGTGCAGTGCGGGCTCCGTAGGGGCACCGGGTGCACCCCGCATACCAGCAGCTCCAGCAGGATCTGCGCCACCTGCCGCCGGGTCAGCGCCACGCGCCAGTCCCGGAGCCCGTTGTCAGTCATGAACAGCTTCAGGGAAGGGACAGACACAGGGAAAGAGGTCAGGTGCGGGGCAGGCAGTGGCACACCCGGCCTGCGCCCCGAGATAAGATGTGGAGGACGCCCTGGGCCAGGAAAGGGT**AGGACAGAGATAGCTGCCCCGGGCGTGGGGCTGGGGAAGGAGGCAGGCGGGAACCCAGCAAGTCATCGCTCCACCTTCTCCACTGTCTCCAACTCTCTGGGATAGTTCTGGCCTACTTTCAGAAGGAATACTCCTCCTGCATGAATTGAGTGCACTGGCAGACTGATTTGTAGCGACCGAGACAGAACCATCGACCTCCCAGGACTATCTGCCCATCCTTGAGGGCTCTGCCCACTTATGACTCTGTGGTAACACCGCTGGCAAAAATAATAGTCAGGGCTTGTGAGATGGCTTAGTGAATAAAGGCATTTGCCACCAAGCCTGACTCCCTGAGTCAACATCGCTAGCATCCACGTTGTGCACGGTGGCGCCAGGTAATTTATAACCTAGGCTGGGCTCACCTGAATGATCTTCCTGCCTCAGACTCCCAAGTGCTGGGACTACAGGTGGGTGCTGCCACACTTCGTTCCATTTATGCAGCATTTGGACGCCCTAGGGACTGCTCTGTCCCCTGTACATGTGACTCAATAAATACACAGAGATTCATGTACTAGCGCTCCCAATTTACAAATCTGAAAATTGAGGCCTGGGTCTCACAACAGGGCAACTGAACACTGGGCCCAGAGCATGAACTGCTCTCTGTGAGGTTTGTTTCTTTGTTTGTTCTTTTGTTTTTGTTTTTGTTTTTGTTTTTGTTTTTGTTTTTTTCAATGTGAGAGCCTCTCACTGGGGGATTCTAGGCAGGGGCTCTACCACTGAGCTACACTCCCAGCCCCTCACTGGGGGATTCTAGGCAGCGCCCCTACCCTATATTACTGTGTCCCTACCTATTGAAATCACAAATCACCTAATGCTTAACATCCCACAGTAGTGTCAGTCCCTAGTGTGAGGTTTGTTTTTTTTTTTCCCCTCCTCCAGACTCAACAACTCTTCCTAGCCATCTTCAAACTCTGGTCTCACTGAAACACAATGCCATTCCCATTTTGATTTCTCCAGATCTTTTTCCCAGTTGCCTGACTCCCACGTAAGACAGTATTCTCAGCCATGTACAGCAATACCCTGGGATGTGCTGGGCACCTAAGAGGCGCTCAGTTAAA**

**Clone 6A**:

GGTGCGTGTTCATGTATAGTTTGGCCACGAACCAGTGGCGGAATCGGACTTGGTTGAGCGCCCCGATGCTGCGGTAAGACGCGTTGAGCAGGACCCCGCTACGCAGAAGTACCGCGCGAGGCACCAGGTAGAGACGTAGCAGCATGGCCAGGGACAGCAACGCCTCGCCTTCGCCCAGGAAGCCCGGCCAGGCCTGTGAGTCTGTGGCCTCCCCCGCCAGGGTGCAGTGCGGGCTCCGTAGGGGCACCGGGTGCACCCCGCATACCAGCAGCTCCAGCAGGATCTGCGCCACCTGCCGCCGGGTCAGCGCCACGCGCCAGTCCCGGAGCCCGTTGTCAGTCATGAACAGCTTCAGGGAAGGGACAGACACAGGGAAAGAGGTCAGGTGCGGGGCAGGCAGTGGCACACCCGGCCTGCGCCCCGAGATAAGATGTGGAGGACGCCCTGGGCCAGGAAAGGGTAGGACAGAGATAGCTGCCCCGGGCGTGGGGCTGGGGAAGGAGGCAGGCGGGAACCC

**Clone 6B**:

GTAGGACAGAGATAGCTGCCCCGGGCGTGGGGCTGGGGAAGGAGGCAGGCGGGAACCCAGCAAGTCATCGCTCCACCTTCTCCACTGTCTCCAACTCTCTGGGATAGTTCTGGCCTACTTTCAGAAGGAATACTCCTCCTGCATGAATTGAGTGCACTGGCAGACTGATTTGTAGCGACCGAGACAGAACCATCGACCTCCCAGGACTATCTGCCCATCCTTGAGGGCTCTGCCCACTTATGACTCTGTGGTAACACCGCTGGCAAAAATAATAGTCAGGGCTTGTGAGATGGCTTAGTGAATAAAGGCATTTGCCACCAAGCCTGACTCCCTGAGTCAACATCGCTAGCATCCACGTTGTGCACGGTGGCGCCAGGTAATTTATAACCTAGGCTGGGCTCACCTGAATGATCTTCCTGCCTCAGACTCCCAAGTGCTGGGACTACAGGTGGGTGCTGCCACACTTCGTTCCATTTATGCAGCATTTGGACGCCCTAGGGACTGCTCTGTCCCCTGTACATGTGACTCAATAAATACACAGAGATTCATGTACTAGCGCTCCCAATTTACAAATCTGAAAATTGAGGCCTGGGTCTCACAACA

**Clone 6C**:

**ACAGAGATTCATGTACTAGCGCTCCCAATTTACAAATCTGAAAATTGAGGCCTGGGTCTCACAACAGGGCAACTGAACACTGGGCCCAGAGCATGAACTGCTCTCTGTGAGGTTTGTTTCTTTGTTTGTTCTTTTGTTTTTGTTTTTGTTTTTGTTTTTGTTTTTGTTTTTTTCAATGTGAGAGCCTCTCACTGGGGGATTCTAGGCAGGGGCTCTACCACTGAGCTACACTCCCAGCCCCTCACTGGGGGATTCTAGGCAGCGCCCCTACCCTATATTACTGTGTCCCTACCTATTGAAATCACAAA**TCACCTAATGCTTAACATCCCACAGTAGTGTCAGTCCCTAGTGTGAGGTTTGTTTTTTTTTTTCCCCTCCTCCAGACTCAACAACTCTTCCTAGCCATCTTCAAACTCTGGTCTCACTGAAACACAATGCCATTCCCATTTTGATTTCTCCAGATCTTTTTCCCAGTTGCCTGACTCCCACGTAAGACAGTATTCTCAGCCATGTACAGCAATACCCTGGGATGTGCTGGGCACCTAAGAGGCGCTCAGTTAAA
