# Supplementary material for: Multiple Mineralocorticoid Response Elements Localized in Different Introns Regulate Intermediate Conductance K+ (Kcnn4) Channel Expression in the Rat Distal Colon
Source: PLoS One. 2014 Jun 5;9(6):e98695. doi: 10.1371/journal.pone.0098695 (PMC4047071; doi:10.1371/journal.pone.0098695)
Supplement: Table S1 — Primer sets used in ChIP. Primer sets are numbered with reference to the genomic DNA block they are directed to amplify. For example, Primer set 1 (forward and reverse primers) RT-PCR amplify approximately 100 bp in DNA block 1, approximately 20 kb upstream of the presumed Kcnn4 transcription start site (TSS). (DOCX) [file pone.0098695.s002.docx]

**Table - S1:** **Primer sets used in ChIP**. Primer sets are numbered with reference to the genomic DNA block they are directed to amplify. For example, Primer set 1 (forward and reverse primers) RT-PCR amplify approximately 100 bp in DNA block 1, approximately 20 kb upstream of the presumed Kcnn4 transcription start site (TSS).

| **Primer set** | **Froward** | **Reverse** |
| --- | --- | --- |
| 1 | 5’-GAAATCCACCTGTCTCTGCC-3’ | 5’-AGCCTGGAAATCACAGTGC-3’ |
| 2 | 5’-TGATGCTCTCCTTCCACAAC-3’ | 5’-GACCTCTAAGTGCTGACACAG-3’ |
| 3 | 5’-ATCCCAAGTTCAAGGTCATCC-3’ | 5’-CTACCTCTCCAGTCCTGTTTG-3’ |
| 4 | 5’-TTCTCTGTGGCTTTGGTCTG-3’ | 5’-ATACCTCTTCCCTCAGACCTC-3’ |
| 5 | 5’-CTAGCAAGGATAGTGGTAACCC-3’ | 5’-TTGGCCTTGAAGTAGCAGTC-3’ |
| 6 | 5’-AAGGTGTTGGGATGTGCTC-3’ | 5’-CTACTGCGGTGAGATATCCAG-3’ |
| 7 | 5’-TGCTTGCCACAAAATCTGATG-3’ | 5’-TGGAGTAAGTGTGAGCATGTG-3’ |
| 8 | 5’-ACCTTTAATCTCAGCACTGGG-3’ | 5’-TTGTCCTGAAACTCGCTCTG-3’ |
| 8B | 5’-TCGTCCGTAGCTCTTCATCTAG-3’ | 5’-CAATCCCTACGTAAACAATTCCAAC-3’ |
| 9 | 5’-ATGTGCTTTGTAGGGTGGAG-3’ | 5’-CTAAGCCTGGTGATCTGTCTTC-3’ |
| 10 | 5’-CTAAAATGCTCCCACCCTCTC-3’ | 5’-GCGACACCATTTCTCCATTTC-3’ |
| 11 | 5’-ACCCTTTCCAGCTTGTCTTC-3’ | 5’-AGCCCTTGGAACTAAACTCAG-3’ |
| 12 | 5’-TCAACGACCTACAATTCCAGC-3’ | 5’-GAGTCCAGAACACAGAAGTACAG-3’ |
| 13 | 5’-GTCTGTAGGAAGCGGATGTAG-3’ | 5’-TCTCCTGTCGGTGTTTGAAC-3’ |
| 14 | 5’-CTTTCTCTAAAGCTCCAGGGC-3’ | 5’-GTACACTGCTACATCCACCTC-3’ |
| 15 | 5’-CCTCCCAGCCTCAATGTATG-3’ | 5’-CAGCATTCACCTAGTCCCTTC-3’ |
| 16 | 5’-GAATCAGCTCTTCCACTCTTCC-3’ | 5’-GCACCTGGAACCGTAGATC-3’ |
| 16B | 5’-CTCCATGCCCCAATCCTC-3’ | 5’-TCTAAGACGGTAGGTGTAGGC-3’ |
| 17B | 5’-CTCCTACATCCAAACTTCCCAG-3’ | 5’-GCCTTGGATCTTATCTGTACCG-3’ |
| 19 | 5’-GCCACCCAAAGTTGATGTTG-3’ | 5’-CTGAGTTCGAATCCCTAGCAC-3’ |
| 20 | 5’-GCATGTTTCCTGTTTCCTGTG-3’ | 5’-CCAAGTAAAACCCCATAACATTCC-3’ |
| 21 | 5’-CTTCCAGCCAAAGTCCCAG-3’ | 5’-ACCTCTCAGTACGCAAATGG-3’ |
| 22 | 5’-CTTGAACTCCCTTGTCCCTAG-3’ | 5’-CCTTCTGATTCTCTTGCCTCC-3’ |
| 23 | 5’-GGTTTACACAGGATGAGGGTC-3’ | 5’-GGGTTTTAGGGTGTCGATGTAG-3’ |
| 24 | 5’-TGGGTGTAGAATTGGAAAAGGG-3’ | 5’-CAGTTTGTCTCAGCCCCTATC-3’ |
| 24B | 5’-AGCGCCATTGTTCAGACAG-3’ | 5’-TCATTGAACCCTGCACCTG-3’ |
| 25 | 5’-TGGAGGGAAGTGAGGATGAG-3’ | 5’-GAGTCTACTTGGGTTATGGCAG-3’ |
| 26 | 5’-TTTAACTGAGCGCCTCTTAGG-3’ | 5’-CTGAAACACAATGCCATTCCC-3’ |
| 27 | 5’-TGCTACGTCTCTACCTGGTG-3’ | 5’-GGTGCGTGTTCATGTATAGTTTG-3’ |
| 27B | 5’-GAGTTGGAGACAGTGGAGAAG-3’ | 5’-AGGAAAGGGTAGGACAGAGATAG-3’ |
| 28 | 5’-ATAGCTCCTGCCAAGTGAAC-3’ | 5’-GAATTCAGCAAGGCAACCAG-3’ |
| 29 | 5’-CCCTGAGAAATGCTGGTATCTG-3’ | 5’-AGGAAGAAGGTGAAGAGGAGAG-3’ |
