# Supplementary material for: Multiple Mineralocorticoid Response Elements Localized in Different Introns Regulate Intermediate Conductance K+ (Kcnn4) Channel Expression in the Rat Distal Colon
Source: PLoS One. 2014 Jun 5;9(6):e98695. doi: 10.1371/journal.pone.0098695 (PMC4047071; doi:10.1371/journal.pone.0098695)
Supplement: Table S2 — Primer sets used for cloning candidate enhancer regions from genomic DNA. (DOCX) [file pone.0098695.s003.docx]

**Table - S2:** Primer sets used for cloning candidate enhancer regions from genomic DNA.

| **Clone** | **Size (bP)** | **Forward primer** | **Reverse primer** |
| --- | --- | --- | --- |
| 1 | 1887 | 5’-GACTAAGCTTGGAGGGAGGCTGGTGTTTTATACCTC-3’ | 5’-TGACAAGCTTTTCGATTGAGATGGAGTTGCGGAGGG-3’ |
| 3 | 675 | 5’-GACTAAGCTTCTGCTCTGGACTGTGAGGCTGCTTG-3’ | 5’-TGACAAGCTTCTACCACTTCCTCTGGCTATGTGCCC--3’ |
| 5 | 1356 | 5’-GACTAAGCTTGGGCAGAATGATGCTCTAGGAGACTT-3’ | 5’-TGACAAGCTTCTGAAACACAATGCCATTCCC-3’ |
| 6 | 1578 | 5’GACTAAGCTTTTTAACTGAGCGCCTCTTAGG-3’ | 5’-TGACAAGCTTGGTGCGTGTTCATGTATAGTTTG -3’ |
| 7 | 1108 | 5’-GACTAAGCTTTGCTACGTCTCTACCTGGTG-3’ | 5’-TGACAAGCTTGAATTCAGCAAGGCAACCAG-3’ |
| 8 | 8, 867 | 5’-GACTAAGCTTATAGCTCCTGCCAAGTGAAC-3’ | 5’-TGACAAGCTTAGGAAGAAGGTGAAGAGGAGAG-3’ |
